# Relative abundance of derelict fishing gear in the Hawaii-based pelagic longline fishery grounds as estimated from fishery observer data

**DOI:** 10.1038/s41598-020-64771-1

**Published:** 2020-05-08

**Authors:** Amy V. Uhrin, William A. Walsh, Jon Brodziak

**Affiliations:** 1 Department of Commerce, National Oceanic and Atmospheric Administration, National Ocean Service, Office of Response and Restoration, Marine Debris Division, Silver Spring, MD 20910 USA; 2 Walsh Analytical Service, Kailua, HI 96734 USA; 3 Department of Commerce, National Oceanic and Atmospheric Administration, National Marine Fisheries Service, Pacific Islands Fisheries Science Center, 1845 Wasp Boulevard, Honolulu, HI 96818 USA

**Keywords:** Environmental sciences, Ocean sciences

## Abstract

Derelict fishing gear (DFG) is abundant across the remote North Pacific Ocean, accumulating in convergence zones that coincide with the fishing grounds of the Hawai’i-based pelagic longline fishery. Longlines are prone to snagging DFG, providing an opportunistic, yet regular, reporting mechanism by longline fishery observers (fishery-dependent data). We apply a zero-inflated negative binomial model previously used to standardize catch per unit effort (CPUE) for bycatch and incidentally caught species in this fishery to estimate DFG relative abundance and qualitative trends within the longline fishing grounds. During 2008–2016, observers reported 1326 marine debris items intercepted by longlines, dominated by DFG at nearly 90%. Modeling results suggest that the relative abundance of DFG has declined ~66% from 2008–2016. DFG standardized CPUE was higher for longlines fished inside the North Pacific Subtropical Convergence Zone (versus outside) and increased moving eastward and northward toward the Great Pacific Garbage Patch. Despite substantially less effort in the shallow-set sector of the fishery (∼60 m depth), DFG standardized CPUE was four-fold greater than that of the deep-set sector (∼250 m) suggesting that marine debris observer reporting focused in this sector may be most effective. Some fishermen voluntarily stow snagged debris; incentivizing at-sea removal may elicit further cooperation.

## Introduction

Derelict fishing gear (DFG) is ubiquitous and abundant across the North Pacific Ocean, originating from several domestic and international large-scale fisheries utilizing the basin. Fishing gear lost, abandoned or discarded anywhere in the North Pacific Ocean may circulate for years in the North Pacific Subtropical Gyre^1,2^. Large amounts of DFG become stranded on shorelines of the Hawaiian archipelago^3,4^, largely as a result of the geographic location of islands in relation to basin-wide circulation patterns. DFG also accumulates within the basin’s subtropical gyres and convergence zones^5–9^.

Recent estimates suggest that derelict fishing nets comprise 46% of debris by mass reported within the boundaries of the Great Pacific Garbage Patch^9^, a known collection of marine debris entrained within the eastern boundary of the North Pacific Subtropical Gyre. Entanglement by DFG is recognized as one of the greatest threats to seabirds, sea turtles, and marine mammals worldwide^10^ and as such it has become a prominent issue that threatens the ecology of the region, especially the Hawaiian archipelago^11^. Quantitative data on DFG is sparse for many regions^12^. The remoteness and vastness of the North Pacific Ocean in particular hampers at-sea detection of this type of surface floating debris, let alone removal. At-sea detection relies heavily on visual observations from vessels and aircraft and remains logistically challenging given the long transit times to reach the area and the high cost and demand for vessel and aircraft time^9,13,14^.

Commercial vessels operating under the purview of the Hawai’i-based pelagic longline fishery exploit the seasonally migrating North Pacific Subtropical Convergence Zone^13,15,16^, an area of both high biological productivity and zone of DFG accumulation which shifts latitudinally between the extremes of 23–37^◦^N^17^. Based on the 0.5^th^ to 99.5^th^ percentiles of reported logbook set locations during 1995–2018, the fishing grounds extend across 4500 km of the western central and eastern North Pacific Ocean from roughly 176°W to 133°W longitude and 5°N to 38°N latitude. Pelagic longlines are comprised of a long (~64–74 km) drifting mainline of thick monofilament that is horizontally suspended in the water column at fixed depths by floats, and from which thousands of baited hooks are attached at intervals via weighted branch lines. Additional information on longline configuration may be found in the Supplementary Text. Longlines are particularly prone to snagging DFG from other fisheries, either while adrift as an active set or during haulback^18^.

The Hawai’i-based pelagic longline fishery is managed by the NOAA Pacific Islands Regional Office and the Western Pacific Regional Fishery Management Council under two sectors defined by depth^19^. Vessels in the deep-set sector target bigeye tuna (*Thunnus obesus*) at roughly 250 m depth deploying upwards of 2300 hooks on average per longline set whereas vessels in the shallow-set sector target swordfish (*Xiphias gladius*) at around 60 m deploying over 1000 hooks per set on average (see Supplementary Tables S1 and S2). The Supplementary Text provides more details about the operational parameters of this fishery. Onboard observations (fishery-dependent data) of species-specific catch and other operational details from vessels participating in this fishery have been made since 1994 as part of the NOAA Pacific Islands Region Observer Program (PIROP)^20^. Currently, PIROP maintains 20% annual observer coverage of deep sector trips and 100% observer coverage of shallow sector trips.

In recognition of the possibility of using longlines to collect data on marine debris, in particular DFG^18^, the NOAA Marine Debris Program together with PIROP developed a standard reporting form for observers to opportunistically record encounters with marine debris during longline fishing operations beginning in 2007^20^. Observations include direct interaction with the longline or vessel, species entanglements, and noteworthy sightings of marine debris at the surface. Observers also record types of DFG (e.g., nets, lines, buoys) and other forms of marine debris encountered. Unlike fishery-independent data where systematic random sampling is employed, fishery-dependent data collected by observers is targeted, spanning the temporal and spatial scales specifically exploited by the fishery, thereby limiting data interpretation to the boundaries of the fishing grounds. However, opportunistic sampling of this nature remains a valuable tool, particularly in undersampled environments. The marine debris data collected by longline observers represent a 9-year time series (2008–2016), one of the longest, continuous at-sea marine debris data sets in the world in one of the most difficult to access areas of the world’s ocean.

In this paper, we apply Generalized Linear Models previously used to standardize catch per unit effort (CPUE) for bycatch and incidental catch in this fishery^21–24^ to estimate the relative abundance and distribution of marine debris intercepted by longline gear over time within the fishing grounds of the Hawai’i-based pelagic longline fishery. We expected marine debris interceptions to be largely dominated by derelict nets and other fishing-associated materials given preliminary examinations of observer data from this fishery^18^. We also considered that interception of marine debris by longlines might exhibit an increase after 2011, following the Tōhoku Japan earthquake and tsunami which generated and released 1.5 million tons of floating debris into the North Pacific Ocean. To our knowledge, this work represents the first comprehensive analysis of fishery-dependent marine debris data gathered as part of a commercial fishery observation program, a rare, novel and entirely opportunistic dataset.

## Results

### Descriptive catch statistics

PIROP fishery observers recorded catch and operational data from 40,572 longline sets during 2911 trips made by Hawai’i-based commercial longline vessels during the period 2008–2016 (see Supplementary Tables S1 and S2). Marine debris was intercepted by 858 sets (2.1% of total observed sets; see Supplementary Tables S1 and S2). A preponderance of longline sets (97.9%) did not snag any marine debris (see Supplementary Tables S1 and S2). Although the number of observed deep sets (N = 32,130) was four-fold greater than shallow sets (N = 8,442), the number of sets with snagged marine debris were similar in both sectors (deep: 418; shallow: 440; see Supplementary Tables S1 and S2).

**Figure 1:**
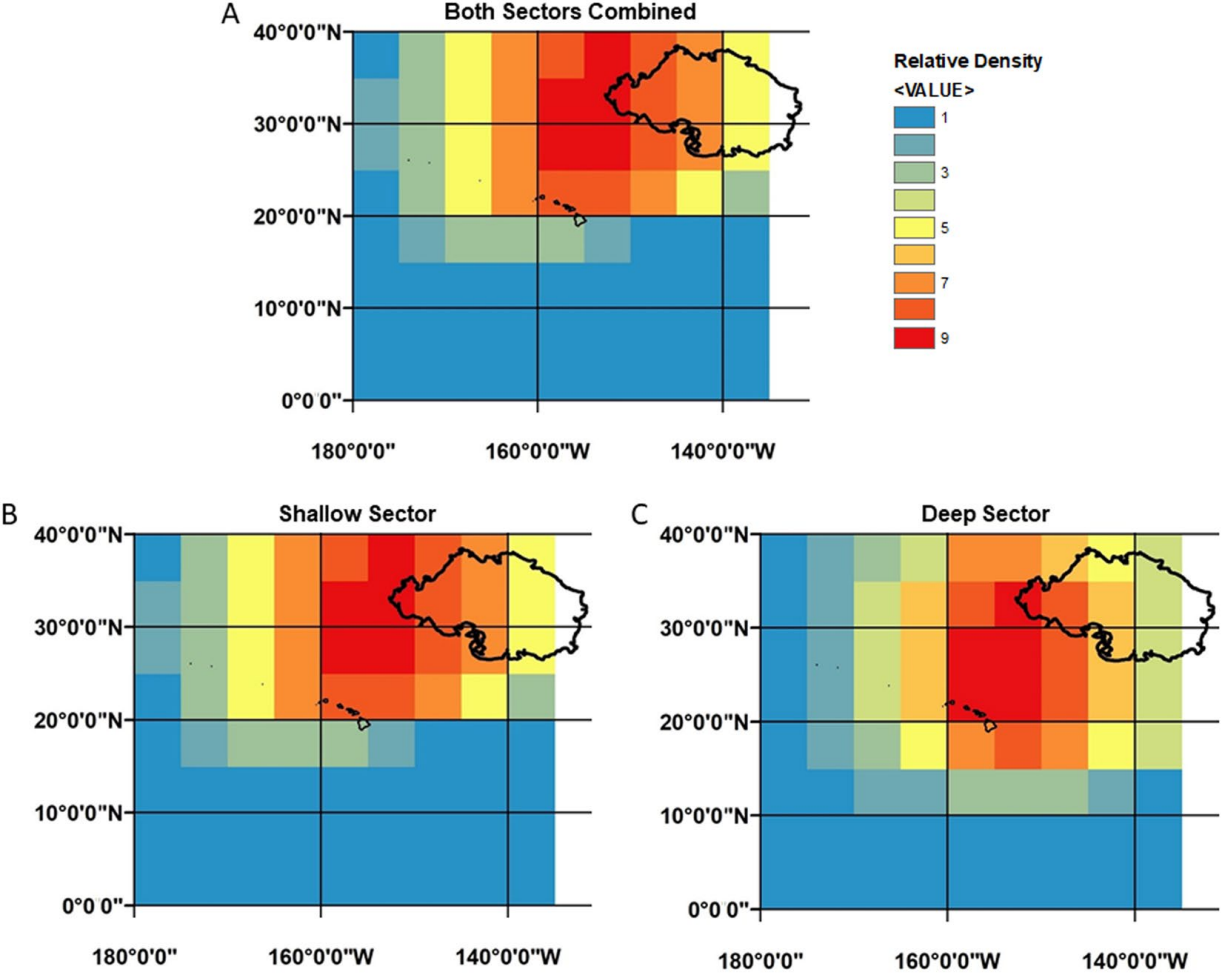
Relative density of marine debris, namely derelict fishing gear, found within 5 × 5 degree raster cells as reported by PIROP fishery observers in the Hawai’i-based pelagic longline fishery during 2008–2016. Relative densities for combined sectors (**A**), shallow (**B**) and deep **(C**) sectors were estimated from original point data representing the haul begin latitude and haul begin longitude of individual longline sets where at least one item of marine debris was intercepted (N = 858). Interpretation of the density surface is qualitative; cooler colors have fewer sets relative to warmer colors. In addition, estimated relative density values are interpreted in relation to the lowest relative density (value = 1, color = blue). Thus, red cells have a longline set density that is nine times as large as the blue cells. The estimated boundary for the Great Pacific Garbage Patch (large black polygon, upper right) is based on plastic mass concentrations for August 2015, as predicted by the models of Lebreton *et al*.^5^. The main Hawaiian Islands (small black polygons, mid-grid) are centered near 19° 34′N and 155° 30′W (Hawai’i) to 21° 54′N and 160° 10′W (Ni’ihau).

Most longline sets with snagged marine debris (97.4% of the total) occurred from 20^◦^ - 40^◦^N, with higher relative densities east of 160^◦^W (Fig. 1A). Above 30^◦^N, higher relative densities of marine debris mainly occurred in sets from the shallow-set sector versus the deep-set sector (336 vs. 75; Fig. 1B), while below 30^◦^N, higher relative densities of marine debris mainly occurred in the deep-set sector versus the shallow-set sector (321 vs. 104 sets; Fig. 1C). Only a few sets reporting marine debris occurred south of 20^◦^N (2.6% of the total) mainly from the deep-set sector (Fig. 1C).

**Figure 2:**
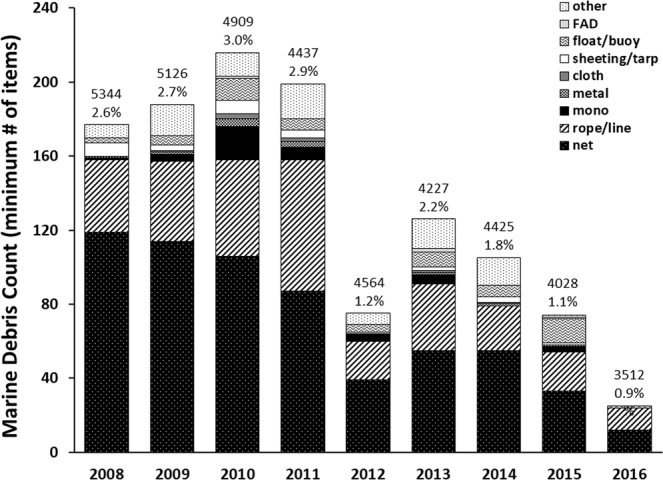
Total minimum number of marine debris items (raw data) by year and type as reported by PIROP fishery observers on board vessels of the Hawai’i-based pelagic longline fishery during 2008–2016. Whole numbers above the bars indicate the number of longline sets observed during that year while underneath is the percent of those sets reported as having snagged marine debris.

A total of 1,326 marine debris items were intercepted by longlines during the period 2008–2016. A frequency distribution of marine debris counts per set indicated that the majority of longline sets captured only one (42.2%) or two (15.4%) items and the maximum number of items captured on a single set was nine. DFG dominated the debris types at nearly 90% of the total (Fig. 2). Most of the DFG consisted of nets (51.8%) and ropes and lines (26.7%) while floats/buoys and monofilament accounted for 4.8% and 3.5%, respectively (Fig. 2). The longlines also intercepted a very small percentage of fish aggregating devices (FAD) (0.3%; Fig. 2). To a lesser extent, the longlines snagged plastic sheeting, cloth, and metal items, as well as an assortment of plastic items including buckets, milk crates, and bait containers, all categorized as other (Fig. 2).

### CPUE standardization

Although all types of snagged debris were reported and included in the model, in the remainder of the paper we reference only DFG given that it represents the bulk of the material (~90%) and is recognized as one of the greatest entanglement threats to wildlife^10^. The set of predictor variables that produced the best fitting zero-inflated negative binomial (ZINB) model included year, quarter, sector, and latitude in the negative binomial counts model and longitude, convergence zone, observer, and sector in the logistic model for zero inflation (see Supplementary Table S3). There was some indication of temporal and seasonal variability in the positive counts (as opposed to zero counts) of DFG. The significant positive coefficients for 2010 and 2011 in the negative binomial count model indicated that positive counts of DFG per set were greater in these years than in 2008 (reference year; Table 1). The significant negative coefficients for 2012 and 2016 indicated that positive counts of DFG per set were lower in these years than in 2008 (Table 1). The only quarterly effect was significantly fewer positive counts of DFG in the third quarter compared to the reference first quarter (Table 1).

**Table 1:** Summary of the zero-inflated negative binomial model for marine debris counts (minimum number of marine debris items) reported from the Hawai’i-based pelagic longline fishery during 2008–2016.

Variable	Estimate (β)	Exp(β)	Std Error	z-value	*p*
*Negative binomial count model*					
Intercept	−11.06	1.57 × 10^−5^	0.55	−20.22	***
2009	0.17	1.18	0.14	1.28	0.202
2010	0.45	1.57	0.14	3.14	**
2011	0.56	1.75	0.15	3.81	****
2012	−0.50	0.61	0.17	−2.90	***
2013	0.25	1.28	0.16	1.57	0.117
2014	−0.001	1.00	0.16	−0.009	0.993
2015	(−0.13)	0.88	0.18	(−0.724)	0.469
2016	−0.46	0.63	0.23	−2.03	*
Quarter 2	−0.09	0.91	0.11	−0.86	0.39
Quarter 3	−0.41	0.66	0.12	−3.33	****
Quarter 4	−0.18	0.83	0.12	−1.48	0.139
Sector	(−0.52)	0.59	0.15	(−3.38)	****
Latitude	0.11	1.12	0.01	7.53	****
*Logistic model for zero inflation*					
Intercept	−5.15	0.01	0.90	−5.76	****
Longitude	−0.05	0.95	0.005	−9.43	****
Convergence zone (within)	−0.83	0.44	0.18	−4.60	****
Observer (high)	−1.58	0.21	0.09	−17.46	****
Sector (deep)	0.82	2.27	0.13	6.34	****

*Significant at p = 0.05, **significant at p = 0.01, ***significant at p = 0.001, ****significant at p = 0.0001. Reference year = 2008, reference quarter = 1.

For every one-degree increase in north latitude, the expected positive counts of DFG per longline set increased by 11.6% (Table 1). For every one-degree decrease in west longitude, we expect to see about a 5% decrease in the odds of an excess zero. Taken together, as one moves northward and eastward in this region, toward the Great Pacific Garbage Patch, the counts of DFG increase. Whether or not fishery effort occurred within the migrating boundaries of the North Pacific Subtropical Convergence Zone (23–37°N) also had a significant effect on DFG counts. More DFG was reported on longline sets fishing within the boundaries of the North Pacific Subtropical Convergence Zone versus sets fishing outside the boundaries (Table 1). Specifically, the odds of excess zero counts decreased by a factor of 0.44 for longline sets occurring within the North Pacific Subtropical Convergence Zone (Table 1). Sector was retained in both models, implying that relative to the shallow sector (reference level), fewer observations of DFG were reported from the deep sector (Table 1). The degree of positive counts of DFG per set was significantly lower in the deep sector by a factor of 0.59 while the odds of having an excess zero increased by a factor of 2.27 (Table 1).

Lastly, the experience level of an observer was important for explaining DFG counts. The odds of excess zero counts decreased by a factor of 0.21 when observers had a high level of experience (≥2.5 years) versus a low level (<2.5 years reference condition; Table 1), meaning that more experienced observers reported more DFG.

### Standardized CPUE trend

**Figure 3:**
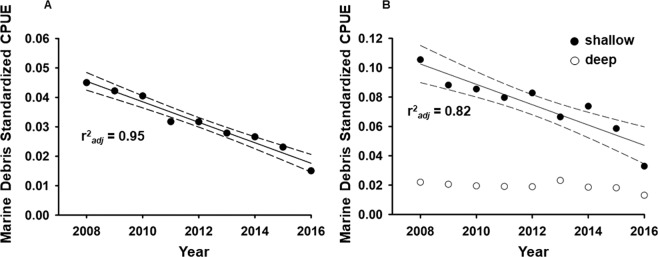
(**A**) Annual standardized CPUE (marine debris items per standardized longline set) in the Hawai’i-based pelagic longline fishery during 2008–1016, all sectors. The solid line represents the time trend regression and the associated 95% confidence intervals are indicated by dashed lines. (**B**) Annual standardized CPUE (marine debris items per standardized longline set) in the Hawai’i-based pelagic longline fishery during 2008–2016, by sector. The shallow sector is indicated by closed circles while the deep sector is indicated by open circles. The solid line represents the time trend regression and with associated 95% confidence intervals (dashed lines).

The annual mean standardized CPUE for DFG (both sectors combined) ranged from 0.015 to 0.045 items per set (Fig. 3A). Mean standardized CPUE from 2008–2010 remained similar peaking at 0.045 items per set (2008), followed by a sudden drop in 2011 to 0.032 items per set (Fig. 3A). Annual mean standardized CPUE continually declined through 2016 (0.015 items per set; Fig. 3A). Linear regression analysis of the standardized CPUE by fishing year indicated that the time trend for DFG (combined sectors) was negative and statistically significant (β = −0.0035, SE = 0.0003, R^2^_adj_ = 0.95, p < 0.0001) with an approximate 66% decline during 2008–2016 (Fig. 3A). Annual standardized CPUE for DFG in the deep-set sector (mean = 0.019, SD = 0.003) was significantly lower than that of the shallow-set sector (mean = 0.075, SD = 0.021) (t_8_ = 8.93, p < 0.001) (Fig. 3B). Linear regression analysis of the standardized CPUE by fishing year and sector indicated that the time trend for DFG in the shallow sector was negative and statistically significant (β = −0.0069, SE = 0.0011, R^2^_adj_ = 0.82, p = 0.0005) with an approximate 69% decline during 2008–2016 (Fig. 3B). The deep sector showed no trend over time (β = −0.0007, SE = 0.003, R^2^_adj_ = 0.34, p = 0.06, Fig. 3B).

## Discussion

Here, we report new information on relative abundance of marine debris, the majority of which was DFG, defined as standardized CPUE, in the fishing grounds of the Hawai’i-based pelagic longline fishery estimated using fishery-dependent data collected by observers for which there are no directly comparable findings. Two other studies have attempted to estimate densities of large marine debris items (e.g., DFG) in this area, using visual observations from aircraft and visual classification of aerial imagery. In a one-month period, Pichel *et al*.^13^ performed three visual observation flights for floating surface debris, each covering an area of approximately 36 km^2^. Comparatively speaking, the flights were concentrated in the western portion of the longline fishery grounds, spanning a rectangular area roughly bounded by 27°−34°N and 169°-155°W^13^. The most common type of debris was DFG (82–88% of sightings per flight), dominated by floats, with a maximum debris density (all items combined) of 2.3 sightings per km^2 ^^13^. Lebreton *et al*.^9^ sampled a 311 km^2^ area for surface floating debris using aerial imagery collected by aircraft operating in an area bounded by 30°-33°N and 144°-135°W (a narrow swath at the easternmost limit of the longline fishing grounds). Semi-automated classification of items sized 10–50 cm and> 50 cm was performed. Using their published percentages of DFG items, and multiplying by the total number of items classified from the image mosaics (1595), we calculated 471 DFG items (29.5%) in an area of 311km^2^ or approximately 1.5 DFG items/km^2 ^^9^. A comparative estimation of DFG items per km^2^ from our longline data is inappropriate given the numerous layers of assumptions involved (e.g., lack of data on length of mainline deployed and longline drift distances limits estimation of area sampled, sampling occurs throughout water column vs. surface only observations). We note that even if all studies were directly comparable, three studies on DFG in the vastness of the North Pacific Ocean only constitutes a beginning toward development of a knowledge base. Consequently, no reliable comparative basis is yet available, and these data await future survey efforts with which contrasts may be made.

The use of fishery-dependent data to quantify marine debris interceptions by longlines is a novel means to estimate the relative abundance and qualitative trends of open ocean marine debris within the fishing grounds of the Hawai’i-based pelagic longline fishery, the majority of which was DFG. We conducted CPUE standardizations as a means to determine operational and spatiotemporal variables important for explaining the relative abundance of DFG and qualitative trend over time within a portion of an oceanic gyre recognized as an accumulation zone. Given the configuration of longline gear (submerged, drifting curtain of hooks), it was not surprising that the most commonly intercepted debris type was DFG from other fisheries, mainly nets and lines, which are large and more easily snagged than smaller, smoother surfaced debris items^18^. Although we were unable to identify the country of origin or assign fishery origination of the DFG, these are likely similar to that of shoreline and nearshore accumulations in the region (bottom and midwater trawls as well as pelagic and coastal driftnets)^3,25^. In the entire data set, only two observations of DFG were specifically identified as longline components, but country of origin was not ascertained.

The ZINB model identified seven predictor variables (year, quarter, sector, latitude, longitude, location within the North Pacific Subtropical Convergence Zone, and observer experience) as the most important in explaining the relative abundance of DFG within the fishing grounds of the Hawai’i-based pelagic longline fishery. Our analysis demonstrates that the number of longline sets having snagged at least one DFG item is higher within the Subtropical Convergence Zone and increases moving eastward and northward from the main Hawaiian Islands toward the Great Pacific Garbage Patch, consistent with ocean circulation and wind drift models^9,15,26–28^.

The number of sets having snagged DFG was nearly equal in both fishery sectors despite significantly greater effort (e.g., number of sets, number of hooks per set) in the deep sector. Additionally, standardized CPUE was four-fold greater in the shallow sector. This may suggest that DFG in this region is either vertically distributed at depths exploited by the shallow sector (around 60 m) and less so as the longlines are deployed deeper (around 250 m), that the geographic distribution of this type of debris at the surface overlaps more frequently with shallow sector longline deployment, or both.

One limitation of using longlines to estimate relative abundance of DFG is not knowing whether debris is intercepted at depth, during the haulback up through the water column (oblique angle), or at the surface. Buoys and floats are typically made of positively buoyant polystyrene while fishing nets and lines are commonly made of high density polyethylene, polypropylene, polyethylene terephthalate or polyamide which are typically neutrally buoyant in seawater. In fact, bundles of derelict fishing nets weighing tens to hundreds of pounds up to multiple tons have been observed floating on the surface of the ocean while having several meters of draft [9, 13, PIROP unpublished data]. Therefore, we speculate that most DFG intercepted by the longlines occurred very close to or at the surface. If DFG capture processes occurred at depth, both sectors employ roughly 20 hour soak times, yielding no sector advantage. If DFG capture processes occurred during retrieval, the average length of a shallow-set longline is shorter and thus should require less haulback time which would reduce exposure to DFG across the depth profile. Even so, it was apparent that the shallow-set sector disproportionately sampled DFG at a higher rate leading us to conclude that there was more DFG present within the surface waters targeted by the shallow sector.

Longlines are specifically configured and deployed to exploit and effectively catch individuals of a target species, one per hook. Hook types do vary between the two sectors. The shallow sector uses 18/0 circle hooks which are slightly larger in width and gape than the 14/0, 15/0, and 16/0 circle hooks predominantly employed in the deep sector but there is limited evidence that this enhances catchability of fish^29,30^ and presumably, debris. Moreover, the large bundles of nets and lines intercepted by the longlines have been observed to snag among multiple hooks or become entangled with the mainline itself. Thus, hook type variation is not likely to influence the capture of DFG given the presence of a kilometers-long curtain of hundreds to thousands of hooks drifting through the water column.

Temporal factors were important in explaining the relative abundance of DFG in the fishing grounds as evidenced by the significance of quarter and more so, year, in the ZINB model. The quarterly effect likely reflects activity patterns in this fishery rather than seasonal distributions of DFG. Both sectors are active during the first and second quarters (January to June), but the shallow sector essentially ceases fishing during the third quarter (July to September) with limited effort in the fourth quarter. Thus, only the third quarter coefficient was significant. The ZINB model estimated that the relative abundance of DFG taken by the Hawai’i-based pelagic longline fishery has significantly declined over time (approximately 66% from 2008 to 2016), a trend largely driven by the steady decline of DFG observed in the shallow sector of the fishery (approximately 69% from 2008 to 2016). We posit that this decline reflects actual declines in the floating DFG population or operational changes in the longline fishery and other fisheries exploiting the North Pacific Ocean although with these data other factors cannot be definitively excluded.

What may be driving this apparent decline in DFG? Despite decades of removal efforts, DFG has continued to strand in large quantities in the nearshore waters and shorelines of the remote NWHI^25,31^. However, the amount removed fluctuates widely on an annual basis depending on the area surveyed and time allotted for removal^31^. The stranded DFG likely represents a legacy of lost gear continuing to circulate in the North Pacific Ocean^1,2^ long after a global moratorium on large-scale, pelagic driftnet fishing was adopted in 1992 (United Nations General Assembly Resolution 46–215). Although resuspension is possible, shoreline and nearshore stranding may remove a portion of DFG from circulation, subsequently reducing the amount available for potential snagging by longlines. Additionally, some longline fishermen retrieve and stow snagged DFG on board and transport it back to port for disposal. Although the amount of DFG removed in this manner has not been quantified, it is possible that over the history of the fishery, stranding and more recently, retrieval practices have helped to decrease the amount of DFG circulating in this region.

Following the 2011 Tōhoku Japan earthquake and tsunami, significant increases in shoreline debris were reported from Hawai’i and along the west coast of North America during 2012–2015^32^ but we did not observe a similar increase in DFG or other marine debris reported from longlines during this time. The majority of floating debris generated by the tsunami consisted of large, high windage items where enough of the item is above the surface of the water (i.e., vessels, docks, building materials, large fishing buoys) to be subjected to wind-driven transport^33^. High windage items did not accumulate in the North Pacific Subtropical Gyre, but rather moved quickly across the basin, stranding on North American shores^33^. Although a substantial number of fishing nets were also reported as lost due to the tsunami, these were predominantly fixed/set nets anchored on the seafloor near the Japan coast and are presumed to have been deposited on the seafloor rather than set adrift^34^.

The tsunami heavily damaged or destroyed several fishing ports in the Tōhoku area, and over 30,000 fishing boats (across all vessel size classes) were lost^34^. The loss of vessels in addition to docking and processing infrastructure has resulted in reduced fishing effort and consequently, the total number of active Japanese commercial longline and purse seine vessels targeting the Pacific Ocean has declined since 2011^34^. Fewer active fishing vessels mean fewer opportunities for lost gear, which may explain to a degree the observed decline in DFG over time in the fishing grounds of the Hawai’i-based pelagic longline fishery. Nonetheless, the actual causes of the modeled decline in DFG standardized CPUE seen in this region have not been identified and cannot be conclusively determined with these data.

The primary uses of fishery-dependent data collected by observers include monitoring protected species interactions in the fishery and assessing stocks of target and bycatch species. Because of the scale of commercial fishing operations, fishery-dependent data often provide greater spatial and temporal coverage than fishery-independent surveys. Marine debris observations conducted in conjunction with the PIROP and the Hawai’i-based pelagic longline fishery provide an opportunistic, but regular mechanism for assessing DFG in these commercial fishing grounds. Here, we have shown changes in relative abundance and qualitative trends for DFG, which is more likely to be intercepted by longlines compared to other forms of debris, and identified categorical factors and covariates that significantly explain DFG relative abundance. The four-fold greater standardized CPUE combined with 100% observer coverage in the shallow sector suggests that marine debris observer reporting focused in this sector may be most effective. Many longline fishermen voluntarily haul and stow snagged debris for disposal in port, effectively removing it from circulation. Thus, future management considerations for the mitigation of marine debris may include incentivizing at-sea removal by fishermen.

## Methods

### Marine debris and fishery data

Catch and operational data collected by PIROP are used for several assessment purposes, including standardization of catch rates for both target and non-target species in the commercial Hawai’i-based pelagic longline fishery^21–24^. Desired catch (i.e., total fish) and operational variables (i.e., # observed vessels, # observed trips, # observer prior trips, # observed sets, # hooks per set, # hooks per float, set begin/end time, set begin/end coordinates, haul begin/end time, haul begin/end coordinates) from the fishery (2008–2016) were extracted from the PIROP database, evaluated using procedures described in^24^ and merged with the MDP database (2008–2016). Analyses were conducted with N = 40,572 observed longline sets. Additional details on data acquisition may be found in the Supplementary Text.

### Catch per unit effort

Catch and effort information from commercial fisheries is the most common source of fishery-dependent (e.g., non-random) data used to develop an index of population abundance for use in stock assessments. Summarized as catch per unit effort (CPUE), this metric only measures the portion of the population susceptible to the fishing gear (not proportional to the total population) within the temporal and spatial scales directly exploited by the fishery. CPUE is standardized to account for the variation in catch rate that cannot be attributed to changes in population size, providing an index of relative abundance. This is achieved by identifying explanatory variables that reduce the unexplained variability in CPUE (response variable) and defining an appropriate statistical distribution for the response variable. Generalized linear models are the most commonly applied method for standardizing catch and effort data in commercial fisheries^35^.

### CPUE standardizations

Standardized CPUE for various bycatch and incidental species catches in the Hawai’i-based pelagic longline fishery are best described using a zero-inflated negative binomial model (ZINB)^22,23^. We anticipated that accidental interception (“catch”) of marine debris (namely, DFG) would also have zero inflation and overdispersion for a number of reasons (see Supplementary Text). Thus, we modeled marine debris interception in this fishery using a ZINB model after confirmatory analyses showed that this model fit the data better than alternative probability models including the Poisson, negative binomial, zero-inflated Poisson and delta-gamma distributions. Full details of generalized linear model fitting procedures using information-theoretic model selection and the theory underlying the ZINB model as related to standardized catch and effort data in this fishery are provided in^22^. The ZINB is a mixture model with two components corresponding to two zero generating processes^36^. The ZINB distribution determines the probability of observing the catch amount (C) as a function of the probability of a false zero catch (*π*), the mean catch (*μ*) and the dispersion (*k*) parameters where the probability of a zero catch is:

1 $$\Pr (C=0)=\,\pi +(1-\pi ){\left(\frac{k}{k+\mu }\right)}^{k}$$

and the probability of a catch amount c is:

2 $$\Pr (C=c|c > 0)=(1-\pi )\frac{(c+k)!}{k!(c+1)!}{\left(\frac{k}{k+\mu }\right)}^{k}{\left(\frac{\mu }{k+\mu }\right)}^{c}.$$

The first process is governed by a binomial distribution (logistic link function) that generates structural zeros wherein zero counts occur because subjects (here, longlines) are simply not at risk for a behavior (i.e., snagging debris). The second process is governed by a negative binomial distribution that generates counts, some of which may be non-zero (positive) and some of which may be zero due to sampling variability (i.e., uncertain, random or chance zeros) and where overdispersion is indicated by a variance to mean ratio greater than unity.

As in other investigations of CPUE in this fishery, individual longline sets were assumed to be independent samples^22,23,37^. However, within a given longline set, marine debris snags could be an individual item of one type, multiple items of various types, or a bundle of multiple items and types ensnared together. Marine debris reporting is not the primary duty of observers. Given the rapid pace at which longline sets are hauled, observers indicated an inability to enumerate every individual item of debris, particularly when a snag involved a large collection of netting and other mixed items. Rather, observers reported the occurrence of a debris interaction, visually assessed the types of marine debris present, and reported a numeric code for every type. Thus, we estimated counts of marine debris items per longline set by summing the number of different types of debris reported over a given set. For example, if a catch of marine debris observed on a set was logged as a tangle of netting, monofilament, and rope (codes 1, 2, 3) then the count of debris would be three items. If a second interaction with debris was observed on the same set, reported as a single item of metal (code 4), then the total count of debris for that set would be four items. We recognize this is a conservative estimate, representing the minimum number of marine debris items snagged per observed longline set.

We considered counts of marine debris per longline set, as estimated above, as the response variable. We evaluated five factor variables as explanatory. Because we were interested in trends over time and differences across season, nine fishing years (2008–2016) and four calendar quarters (1^st^ – 4^th^) were included. We considered fishery sector (deep-set vs shallow-set) as this represents the basis for management and yields insight as to potential depth distribution of debris. Two levels of observer experience were included (low vs high) as the experience level of individual observers may influence reported marine debris. Observers averaging 2.5 years of experience prior to 2008 were considered ‘high’ observers and all others were considered ‘low’. We considered geographic location relative to the boundaries of the North Pacific Subtropical Convergence Zone as a two-level factor, inside (23–37°N) versus outside the defined boundaries^17^. Two additional factor variables, five levels of incident type and eight levels of fishing region were tested in preliminary analyses but did not meet the criteria for inclusion as explanatory variables. We evaluated two continuous variables as explanatory. These included the latitude and longitude, in decimal degrees, of the longline set as it was deployed which represented the spatial distribution of marine debris. We also considered the total catch of all species as their presence on the longline gear may reduce marine debris snags (by taking up available hooks) and vice versa but total catch did not meet the criteria for inclusion as an explanatory variable. One additional continuous variable, soak duration, was tested in preliminary analyses but did not meet the criteria for inclusion.

We included number of hooks per set as an offset term to adjust for differences in effort across sets and fishery sectors. The model was fitted by stepwise selection beginning with the factor variables, followed by the continuous variables. Interactions were not included because the number of empty factor combinations was considered excessive^38^. We assessed the relative importance of each explanatory variable via reductions in the Akaike Information Criteria (AIC) following each variable entry as described in^22^. We computed chi-square tests at each entry stage to evaluate the statistical significance of explanatory variables. Resulting parameter estimates are given on the log (negative binomial count model) and logit scales (logistic model for zero inflation), therefore we exponentiated the parameter estimates to return the response variable to its original count scale. Exponentiated parameter estimates reflect counts and odds ratios for the negative binomial and logistic models, respectively. Because the log link function used in the negative binomial model causes continuous variables (i.e., latitude, longitude) to have a non-linear relationship with the response variable, we cannot use the interpretation above as for factor variables. For continuous variables, we calculated the percent change in expected counts using the following equation:

$$Percent\,change=100\times [exp(\beta \,\times \,\Delta )-1]$$

where β is the parameter estimate, and Δ is the amount of change in the predictor (i.e., for a one-unit change in latitude or longitude, Δ = 1). Model diagnostics included the reduction in AIC by each explanatory variable, the median residual, and plots of Pearson residuals.

Standardized CPUE was calculated using the ZINB model by setting factor and continuous variables at their mean values and then applying the fitted effect coefficients to predict the standardized annual CPUE time series mean and variance^24^. The ZINB model was computed in R (version 3.4.1) using the ‘zeroinfl’ function in the package ‘pscl’^39^. Chi-square tests employed a *p* < 0.05 significance criterion. Differences in marine debris standardized CPUE between the two sectors was evaluated using a t-test in SigmaPlot (version 12.5) with years as replicates.

### Standardized CPUE time trend

We used linear regression on the annual mean standardized CPUE to evaluate the marine debris trend for both sectors combined and for each sector individually. Linear regression was performed in SigmaPlot (version 12.5).

### Spatial distribution of marine debris

The Point Density process in ArcMap™ (version 10.2.1) was used to calculate the the density of longline sets having reported at least one item of marine debris (N = 858) and create a surface to show where longline sets are concentrated. Longline set densities were estimated within 5 ×5 degree raster cells (search area) from original point data representing the haul begin latitude and haul begin longitude of each longline set. Longline sets falling within the search area were summed, then divided by the search area size to estimate each cell’s longline set density value.

## Supplementary information

Supplementary Information

## Data Availability

The fisheries operations data that support the findings of this study are available from the NOAA Pacific Islands Regional Office (PIRO) but restrictions apply to the availability of these data per NOAA Administrative Order 216-100, Protection of Confidential Fisheries Statistics. Access is restricted to authorized individuals who have submitted a signed non-disclosure agreement (annually renewed) to Stefanie Dukes, NOAA PIRO Sustainable Fisheries Division (stefanie.dukes@noaa.gov). Data must not be shared with non-authorized individuals. These data were made available to the authors for analytical purposes subject to the authors abiding by the NOAA Statement of Non-Disclosure of Confidential Data. Because of the confidentiality of fisheries operations data, individual vessel identifiers cannot be attached to any individual data items which are made public. Vessel identifiers include vessel name or permit number; individual data items include fishing location, catch, and effort. Vessels could be differentiated by random identifiers for some purposes. The dataset generated for this analysis (which includes both fisheries operations data and data on marine debris counts) is available directly from the corresponding author (Amy V. Uhrin, amy.uhrin@noaa.gov) upon reasonable request and with permission of the NOAA PIRO, noting that vessel identifiers will be replaced with random identifiers and individual vessel locations will be withdrawn.
