# Supplementary material for: Relative abundance of derelict fishing gear in the Hawaii-based pelagic longline fishery grounds as estimated from fishery observer data
Source: Sci Rep. 2020 May 8;10:7767. doi: 10.1038/s41598-020-64771-1 (PMC7210986; doi:10.1038/s41598-020-64771-1)
Supplement: Supplementary file 1 — Supplementary Information [file 41598_2020_64771_MOESM1_ESM.pdf]

Supplementary Information for

Relative abundance of derelict fishing gear in the Hawaii-based pelagic longline fishery grounds as estimated from fishery observer data

Amy V. Uhrin[1]*, William A. Walsh[2], Jon Brodziak[3]

[1]Department of Commerce, National Oceanic and Atmospheric Administration, National Ocean Service, Office of Response and Restoration, Marine Debris Division, Silver Spring, MD 20910 USA.

[2]Walsh Analytical Service, Kailua, HI 96734 USA.

[3]Department of Commerce, National Oceanic and Atmospheric Administration, National Marine Fisheries Service, Pacific Islands Fisheries Science Center, 1845 Wasp Boulevard, Honolulu, HI 96818 USA.

*Corresponding author: Amy V. Uhrin

E-mail: amy.uhrin@noaa.gov

Tel.: 240-533-0426

Fax: 301-713-4389

This PDF file includes:

Supplementary text

Supplementary Tables S1 to S3

Supplementary References

SI Text

Methods

Fishery description

Catches from the Hawai'i-based pelagic longline fishery account for 90% of commercial pelagic landings in Hawai'i with over 33 million pounds landed in 2016 having an ex-vessel value exceeding $100 million [1]. Vessels in the deep-set sector target bigeye tuna (*Thunnus obesus*) at roughly 250 m deploying 20-32 hooks per float

(mean: 26.8) whereas vessels in the shallow-set sector target swordfish (*Xiphias gladius*) at around 60 m deploying two to six hooks between floats (mean: 4.3) [2]. Shallow-set vessels typically deploy longline gear around sunset using over 1000 hooks per set on average (range: 915.6 - 1102.8) and their activity is concentrated at relatively high latitudes (ca. 27-37°N) during the first and fourth quarters of the calendar year. Deep-set sector vessels deploy gear after dawn using upwards of 2300 hooks on average per longline set (range: 2255.5 - 2544.7) and operate southward toward the equator with continuous and widespread fishing effort throughout the year. Soak times for both sectors are on the order of 20 hours with slight variation [2].

Longline gear configuration

The submerged mainline does not extend as a straight line but rather assumes the shape of a catenary due to the weight of the branch lines. Thus, the depth of the deepest settled hook and the angle of the catenary depend on the number of hooks between floats. Shoaling of the mainline also occurs in response to environmental factors such as wind stress, surface current velocity, and current shear [2]. Without the aid of depth recording devices attached to the gear and subsequent use of catenary geometry, it is difficult to accurately estimate the total length of a deployed longline and the fishing depth. Bigelow et al. [2] experimentally deployed temperature-depth recorders to derive mean mainline lengths of 74.5 km (range: 18.5-111.1) and 64.4 km (range: 27.8-111.1) for the shallow and deep sectors, respectively. In the same study, median depth of the settled deepest hook was estimated to be 59.8 m and 248 m for the shallow and deep sectors, respectively [2].

Regulations enacted in 2004 to reduce sea turtle interactions with longline gear in the shallow sector where such interactions are more common, require the use of circle hooks with minimum size of 18/0 and no more than a 10° offset with whole fish bait. Since 2005, fishermen in the deep sector have progressively been voluntarily switching to circle hooks of size 14/0, 15/0, or 16/0 which represents the greatest proportion of hook types used (30-40%) [2]. A very small proportion of fishermen continue to use Japanese-style tuna hooks or 18/0 circle hooks and some fishermen use a combination of the above types [2].

Pacific Islands Region Observer Program data

In 1994, NOAA's Pacific Islands Region Observer Program (PIROP) initiated onboard observations of species-specific catch and other operational details from vessels participating in the Hawai'i-based pelagic longline fishery as a means of measuring interaction rates between longline gear and protected and endangered species. Observers record species-specific catch tallies from each longline set together with a suite of operational parameters utilizing protocols established by the NOAA Fisheries Pacific Islands Regional Office [4].

PIROP has maintained 17-22% annual observer coverage of deep-set sector trips since 2001 and 100% observer coverage of the shallow-set sector since 2005. The main priority of PIROP observers is the collection of information pertaining to incidental interactions of protected species with commercial longline fishing activities (i.e., longline set and haul). The Hawai'i Longline Observer Program Field Manual [3] describes the full complement of protocols and required forms/logs. At the conclusion of a trip, observers report to the PIROP for debriefing and enter data into the electronic Longline

Observer Database maintained by NOAA's Pacific Islands Regional Office. The catch and operational data collected by PIROP are used for several assessment purposes [4], including standardization of catch rates for target species (tuna, swordfish), bycatch (i.e., sharks, seabirds, sea turtles) and incidental catch [5-9].

Marine Debris Program data

In 2007, PIROP observers began using the Marine Debris Encounter Report [3] to record items of marine debris observed during longline hauls. Upon observing debris, observers recorded the trip number, date, time, and the GPS coordinates. Items were assigned a debris type using the following numeric codes: 1) net, 2) rope/line, 3) monofilament, 4) metal, 5) cloth, 6) plastic sheeting/tarp, 7) floats/buoys, 8) fish aggregating device, 9) other, 10) lumber, and 11) natural debris (i.e., logs). Optional descriptive information was also recorded as well as whether the debris was brought on board. At the conclusion of a trip, the Marine Debris Encounter Reports were provided to the MDP whose staff entered the data into an electronic database maintained by the MDP.

CPUE standardizations

Standardized CPUE for various bycatch and incidental species catches in the Hawai'i-based pelagic longline fishery are best described using a zero-inflated negative binomial model (ZINB), a result of excess zero catches as well as overdispersion (aggregated distribution) in both positive and zero catches [6, 9, 10]. We anticipated that accidental interception ("catch") of marine debris would also have zero inflation and overdispersion for a number of reasons. It is possible lack of physical overlap between the actively fished longline and floating/submerged marine debris results in

“noncatchability” of some debris present in the water column and at the surface but not distributed in the depth range or surface area exploited by this fishery, leading to extra zero observations. Secondly, the primary duty of fishery observers is to sample for target and protected species. Consequently, observers may not be perfectly recording marine debris due to competing priorities [6], resulting in extra zero observations. Derelict fishing nets capture and retain smaller debris items, forming large bundles with patchy distributions and affected by larger scale and numerous spatially and temporally variable mesoscale eddies spurring off the North Pacific Subtropical Convergence Zone [11]. These eddies may accumulate or repel marine debris depending on direction of rotation [12]. Thus, a high probability of zero marine debris interactions may occur if vessels do not encounter these accumulations or eddies (but the interception rate may be very high when an encounter occurs).

Supplementary Table S1. Summary of marine debris data from the shallow-set sector of the Hawai'i-based pelagic longline fishery as reported by PIROP fishery observers during 2008-2016. Observer prior trips (experience), hooks per set, and hooks per float are presented as means ± one standard error (SE).

Year	# observed vessels	# observed longline trips	# observed longline sets	# observers	# observer prior trips (SE)	# trips with marine debris	# sets with marine debris (% of total sets observed)	# hooks per set (SE)	# hooks per float (SE)
2008	26	86	1475	46	16.5 (4.0)	39	90 (6.1%)	915.6 (7.5)	4.3 (0.6)
2009	28	104	1635	54	16.0 (3.7)	37	81 (4.9%)	964.5 (7.8)	4.8 (0.1)
2010	26	93	1561	52	14.4 (3.9)	37	81 (5.2%)	978.6 (8.1)	4.8 (0.1)
2011	17	52	928	37	16.9 (5.6)	21	50 (5.4%)	1013.5 (11.1)	4.6 (0.05)
2012	16	55	913	38	18.6 (4.9)	14	38 (4.2%)	1098.0 (11.6)	5.2 (0.05)
2013	10	28	455	21	24.1 (8.2)	12	26 (5.7%)	1094.3 (14.1)	5.21 (0.1)
2014	14	41	643	30	24.2 (6.4)	20	37 (5.7%)	1084.5 (16.2)	5.0 (0.04)
2015	16	31	491	26	21.2 (6.9)	10	25 (5.1%)	1102.8 (14.5)	5.1 (0.1)
2016	7	19	341	14	19.9 (12.1)	5	12 (3.5%)	1102.6 (16.6)	4.8 (0.1)
**Total**	**160**	**509**	**8442**	**318**		**195**	**440**		

Supplementary Table S2. Summary of marine debris data from the deep-set sector of the Hawai'i-based pelagic longline fishery as reported by PIROP fishery observers during 2008-2016. Observer prior trips (experience), hooks per set, and hooks per float are presented as means ± one standard error (SE).

Year	# observed vessels	# observed longline trips	# observed longline sets	# observers	# observer prior trips (SE)	# trips with marine debris	# sets with marine debris	# hooks per set (SE)	# hooks per float (SE)
2008	115	291	3869	84	12.2 (2.8)	31	47 (1.2%)	2255.5 (11.8)	26.0 (0.1)
2009	117	255	3491	81	13.0 (3.1)	35	59 (1.7%)	2250.6 (12.3)	25.7 (0.1)
2010	108	245	3348	92	10.9 (3.0)	37	65 (1.9%)	2276.4 (12.7)	25.5 (0.1)
2011	116	260	3509	81	11.3 (3.1)	45	81 (2.3%)	2346.9 (13.0)	24.9 (0.1)
2012	121	273	3651	77	14.7 (3.4)	13	15 (0.4%)	2409.8 (13.3)	25.1 (0.1)
2013	122	282	3772	73	16.9 (3.5)	43	67 (1.8%)	2440.3 (13.1)	25.1 (0.1)
2014	125	279	3782	74	18.7 (3.9)	35	43 (1.1%)	2523.1 (13.3)	25.0 (0.1)
2015	123	277	3537	82	17.5 (4.1)	17	21 (0.6%)	2520.9 (14.6)	25.0 (0.1)
2016	116	240	3171	59	22.8 (5.4)	15	20 (0.6%)	2544.7 (13.3)	24.7 (0.1)
**Total**	**1063**	**2402**	**32,130**	**703**		**271**	**418**		

Supplementary Table S3. Summary of stepwise variable selection results for a zero-inflated negative binomial model of marine debris counts (minimum number of marine debris items per set), namely derelict fishing gear, reported from the Hawai'i-based pelagic longline fishery during 2008-2016. The summary includes degrees of freedom (df) associated with each variable, Akaike Information Criterion (AIC), the reduction in AIC (ΔAIC), the reduction in AIC per degree of freedom (ΔAIC/df), the median residual from the fitted model, and the maximum likelihood estimate of the overdispersion parameter (*k*).

Parameter	df	AIC	ΔAIC	ΔAIC/df	Median residual	k
Negative binomial count model						
Intercept	1	10439.22	.	.	-0.1162	0.1912
Year	8	10328.39	110.82	13.85	-0.1140	0.0215
Quarter	3	10218.97	109.42	36.47	-0.1183	0.1527
Sector	1	9628.69	590.28	590.28	-0.1174	0.0414
Latitude	1	9293.10	335.60	335.60	-0.1145	0.0529
Logistic model for zero inflation						
Longitude	1	9206.19	86.91	86.91	-0.1118	0.1498
Convergence zone	1	9184.95	21.24	21.24	-0.1130	0.1824
Observer	1	8819.69	365.26	365.26	-0.0966	0.5098
Sector	1	8786.13	33.56	33.56	-0.0925	0.8377

Supplementary References

1. Western Pacific Regional Fishery Management Council. Annual stock assessment and fishery evaluation report for U.S. Pacific Island pelagic fisheries 2018, http://www.wpcouncil.org/wp-content/uploads/2019/07/Pelagic-FEP-SAFE-Report-2018-Optimized-v4.pdf (2018)
2. Bigelow, K., Musyl, M. K., Poisson, F., & Kleiber, P. Pelagic longline gear depth and shoaling. *Fish. Res.* **77**, 173-183 (2006).United States Department of Commerce. Fisheries off West Coast states and in the western Pacific; highly migratory species fisheries.
3. Pacific Islands Region Observer Program. Hawai'i longline observer program field manual version LM.17.02, https://www.fisheries.noaa.gov/pacific-islands/fisheries-observers/pacific-islands-region-observer-resources (2017)
4. Walsh, W. A., & Brodziak, J. Applications of Hawai'i Longline Fishery Observer and Logbook Data for Stock Assessment and Fishery Research, NOAA Technical Memorandum NOAA-TM-NMFS-PIFSC-57, https://repository.library.noaa.gov/view/noaa/14384 (2016)
5. McCracken, M. L. Modeling a very rare event to estimate sea turtle bycatch: lessons learned. NOAA Technical Memorandum, NOAA-TM-NMFSPIFSC-3, https://pifsc-www.irc.noaa.gov/library/pubs/tech/NOAA_Tech_Memo_PIFSC_3.pdf (2004)

6. Brodziak, J. & Walsh, W. A. Standardizing catch rates of bycatch species using multimodel inference: A case study of oceanic whitetip shark in the Hawai'i longline fishery. *Can. J. Fish. Aquat. Sci.* **70**, 1723–1740 (2013)
7. Gilman, E., Chaloupka, M., Peschon, J., & Ellgen, S. Risk factors for seabird bycatch in a pelagic longline tuna fishery. *PLoS ONE* **11(5)**, e0155477 (2016)
8. Swimmer, Y., Gutierrez, A., Bigelow, K., Barceló, C., Schroeder, B., Keene, K., Shattenkirk, K. & Foster, D. G. Sea turtle bycatch mitigation in U.S. longline fisheries. *Front. Mar. Sc*i. **4**, 260 (2017)
9. Walsh, W. A. & Brodziak, J. Billfish CPUE standardization in the Hawai'i longline fishery: model selection and multimodel inference. *Fish. Res.* **166**, 151-162 (2015)
10. Zuur, A., Ieno, E. N., Walker, N. J., Saveliev, A. A. & Smith, G. M. Zero-truncated and zero-inflated models for count data in *Mixed Effects Models and Extensions in Ecology with R*. (eds. Gail, M., Krickeberg, K., Samet, J. M., Tsiatis, A. & Wong, W.) 261-294 (Springer Science + Business Media, LLC, 2009).
11. Roden, G. I. Subarctic-subtropical transition zone of the North Pacific: largescale aspects and mesoscale structure in *Biology, Oceanography and Fisheries of the North Pacific Transition Zone and the Subarctic Frontal Zone,* NOAA Technical Report NMFS 105. (ed. Wetherall, J. A.) 1-38 (US Department of Commerce, 1991).
12. Howell, E. A., Bograd, S. J., Morishige, C., Seki, M. P. & Polovina, J. J. On North Pacific circulation and associated marine debris concentration. *Mar. Poll. Bull.* **65**, 16-22 (2012).
